# Electrical Impedance Tomography for Robot-Aided Internal Radiation Therapy

**DOI:** 10.3389/fbioe.2021.698038

**Published:** 2021-06-21

**Authors:** Hao Tan, Carlos Rossa

**Affiliations:** Faculty of Engineering and Applied Science, Ontario Tech University, Oshawa, ON, Canada

**Keywords:** electrical impedance tomography, brachytherapy needles, high dose rate brachytherapy treatment, modified newton raphson, robotics, imaging

## Abstract

High dose rate brachytherapy (HDR) is an internal based radiation treatment for prostate cancer. The treatment can deliver radiation to the site of dominant tumor growth within the prostate. Imaging methods to delineate the dominant tumor are imperative to ensure the maximum success of HDR. This paper investigates the feasibility of using electrical impedance tomography (EIT) as the main imaging modality during robot-aided internal radiation therapy. A procedure utilizing brachytherapy needles in order to perform EIT for the purpose of robot-aided prostate cancer imaging is proposed. It is known that cancerous tissue exhibits different conductivity than healthy tissue. Using this information, it is hypothesized that a conductivity map of the tissue can be used to locate and delineate cancerous nodules via EIT. Multiple experiments were conducted using eight brachytherapy needle electrodes. Observations indicate that the imaging procedure is able to observe differences in tissue conductivity in a setting that approximates transperineal HDR and confirm that brachytherapy needles can be used as electrodes for this purpose. The needles can access the tissue at a specific depth that traditional EIT surface electrodes cannot. The results indicate the feasibility of using brachytherapy needles for EIT for the purpose internal radiation therapy.

## 1. Introduction

In Canada, prostate cancer is the most common form of cancer among men with 22,900 in 2019 alone (Canadian Cancer Society, 2019). On average, 63 men are diagnosed with prostate cancer everyday in the country. When the cancer is detected late and the growth is determined to be metastatic and has spread to other major organs, 3 out these 4 men may succumb to the cancer (Canadian Cancer Society, 2019; Cancer.net Editorial Board, 2019). Depending on the severity and stage of the cancer, different treatments may be prescribed. These can range from radiation or hormone therapy, cryotherapy to prostatectomy (American Cancer Society, 2020). In terms of radiation based treatment, radiation may be administered using either an internal or external radiation source. Brachytherapy is an internal based radiation treatment that enables precise delivery of radiation to target specific cancer cells. Using long needles, radiation is delivered to target cancer cells through the perineum. There are two types of brachytherapy: permanent (lose dose rate, or LDR) brachytherapy, and temporary (high dose rate, or HDR) brachytherapy. For LDR, small radioactive pellets (such as iodine-125 or palladium-103) are placed inside the needles and delivered into the prostate gland permanently. For HDR, radioactive iridium-192 or cesium-137 is delivered into the patient's body. A hollow needle is inserted into the prostate and radiation is delivered for ~5–15 min before the needles and the radiation source are removed (American Cancer Society, 2020).

Temporary brachytherapy, HDR, offers better clinical control over the dose administrated to the targeted area when compared to LDR (Prostate Cancer Canada, 2020). In turn, this reduces the negative impact of radiation on surrounding areas, such as the rectum and urethra. Moreover, HDR offers lower radiation exposure for the clinical staff when compared to LDR. Over the past 20 years, HDR has been increasingly implemented as a result of these factors (Kent, 2020).

Although brachytherapy radiation is often imposed upon the entire prostate gland, in many cases, a dominant tumor growth exists within a certain area of the gland. The dominant tumor is often the epicenter and driver of the malignant cancer growth. One benefit of performing HDR is that specific areas in the prostate can be targeted and this focal area can benefit from an escalated radiation dose to better combat the cancer locally (American Cancer Society, 2020; Prostate Cancer Canada, 2020). Focusing the radiation treatment on a specific focal area can reduce adverse side effects such as urinary incontinence, rectal symptoms, erectile, and prostate gland dysfunction that may come with traditional whole-prostate gland radiation treatment (Orton, 2001). To this end, imaging methods that are able to delineate the dominant prostate foci are imperative as it gives the clinical staff critical information regarding the location of the dominant prostate foci (Bauman et al., 2013). Imaging the intraprostatic tissue also plays an important role in robot-aided brachytherapy, where robotic agents are used to steer brachytherapy needles toward the tumor while compensating for needle deflection and tissue deformation. For comprehensive review on the topic, the reader is referred to Rossa and Tavakoli (2017). In these systems, a robotic system acts as a needle holder that can move the needle laterally or rotate it at its base to assist with insertion (Schneider et al., 2004; Wei et al., 2004; Fichtinger et al., 2008; Salcudean et al., 2008; Bassan et al., 2009; Kobayashi et al., 2009; Adebar et al., 2011; Hungr et al., 2012; Long et al., 2012; Bebek et al., 2013; Hendrick et al., 2015; Plitea et al., 2015; Vitrani et al., 2016; Mascarenhas et al., 2017; Rossa and Tavakoli, 2017; Wang et al., 2019; Fanhao et al., 2020).

Contemporary imaging strategies for prostate cancer include ultrasound, elastography, fluorodeoxyglucose with positron emission tomography, as well as magnetic resonance imaging (MRI) (Mansbridge et al., 2019). Grayscale or B-Mode ultrasound is the most commonly used imaging technique for HDR brachytherapy (Banerjee et al., 2017). Although B-mode ultrasound is excellent at delineating the general prostate anatomy, it has its limitations when applied to prostate cancer imaging. Oftentimes, nonmalignant cell tissue, such as inflammation, can appear hypoechoic. This results with the nonmalignant healthy tissue appearing darker than the surrounding tissues, which may lead to misdiagnosis of prostate cancer. On the other hand, studies show that up to 60% of morphological suspicious lesions may be reported as benign while inspected using ultrasound imaging, even though the nature of the tissue may be malignant (Sarkar and Das, 2016). In addition, gray scale ultrasound may sometimes portray early stage carcinoma as isoechoic. This results with the carcinoma appearing similar to surrounding healthy tissue, with no clear distinction (Porter and Banerji, 2016; Ebeid and Elshamy, 2018).

Aside from ultrasound, MRI has also been implemented frequently in terms of prostate cancer diagnosis (Sarkar and Das, 2016). However, like ultrasound imaging, MRI has its own limitations that prevent it from being employed universally. The main drawback of MRI is the relatively higher cost and time it takes to perform the imaging procedure (Kim et al., 2018). The need for an accurate and cost effective imaging techniques has been increasing and is prominent in contemporary HDR brachytherapy.

An imaging technique for brachytherapy applications that has not been explored thoroughly in the literature is the use of electrical impedance tomography (EIT). EIT is a non-invasive and inexpensive way of medical imaging when compared to methods like MRI, X-Ray, and ultrasound (Hughes et al., 1994; Davidson et al., 2012). The main advantages of implementing EIT are unarguably its relatively fast and cost beneficial process as well as its high contrast images. The use of EIT has been well-documented over the years, and its applications include but are not limited to: cranial imaging of newborns, hyperthermia treatment, breast imaging, and among many others (Zhai et al., 2008, 2010; Ferraioli et al., 2009; Blankman et al., 2013; Hough et al., 2014; Murphy et al., 2017; Zuluaga-Gomez et al., 2019; Chen et al., 2020). The process of EIT involves placing electrodes around the periphery of the medium of interest. Electrical current is induced into the medium via the peripheral electrodes and the resulting voltage values are recorded (Seo, 2012). Using the recorded voltage values, the internal resistance distribution of the medium is then reconstructed and an image is produced to portray the anatomy of the medium. It has been well-documented that cancerous tissue exhibit significantly different conductivity values than healthy tissue (Wan et al., 2013; Balidemaj et al., 2016). Using the high contrast imaging nature of EIT, the conductivity difference between cancerous tissue and its surrounding healthy tissue can be displayed on an image.

Typically for EIT, external electrodes are placed on the periphery of the medium of interest. The voltages are then measured directly on the surface (Zhou et al., 2018). Although this approach is often sufficient in obtaining general information regarding the tissue it may not be able to discriminate between tissue conductivity deep underneath the surface. Tissue variations can exist well below the skin surface and electrodes may not able to detect this change in conductivity (Meroni et al., 2017). This is especially the case for prostate cancer (Meir and Rubinsky, 2015). The prostate is located deep underneath the skin surface from all sides and is neighbored by several organs of significant mass (i.e., bladder). The physical distance and the neighboring organs can all attribute to noise in surface electrode measurements. In contrast, research has been conducted into utilizing internal electrodes that are placed within the object of interest, much like needles, in order to perform the imaging procedure (Halter et al., 2011; Mishra et al., 2011, 2013; Wan et al., 2013; Fukushima et al., 2015; Meir and Rubinsky, 2015; Meroni et al., 2017; Kwon et al., 2018; Park et al., 2018; Abbasi et al., 2020). With the use of needle probes, the voltage measurements can be taken deep underneath the skin surface to the exact depth of the suspicious tissue under inspection. This concept has been explored in applications like electrolysis and general biopsy and tissue identification procedures. In the context of EIT for prostate cancer, the electrodes are grouped together or placed at a single location (Halter et al., 2011; Mishra et al., 2011, 2013; Wan et al., 2013; Park et al., 2018; Abbasi et al., 2020). There has not been research into conducting EIT using multiple brachytherapy needles fabricated for the purpose of HDR and prostate cancer and application of image-guided robotic interventions.

In this paper, we postulate brachytherapy needles may be used as electrodes for EIT and thereby one can obtain a map of the internal conductivity of the tissue to delineate intraprostatic tissue and use this information in robot-aided interventions. This paper explores the early steps on the feasibility of this concept and will focus on creating the EIT images. In future work, these images may be used to provide feedback to a robotic manipulator during HDR. Eight standard 18-gauge brachytherapy needles are used as EIT electrodes to deliver current to the tissue while the induced voltage is measured via an impedance spectroscopy analyzer. With this approach, EIT can be performed under the surface of the tissue, directly or near the tumor. The high contrast imaging nature of EIT can improve radiation dose delivery in two major ways. First, the high spatial resolution of the images can distinguish the prostate from nearby structures. Second, the high contrast images can define the dominant region of the cancer growth within the prostate. Knowing the dominant region of cancer growth, radiation delivery can move away from whole gland treatment to target a specific area instead. Accurate delineation of the malignant lesion would allow radiation oncologists to escalate the radiation level in the dominant growth while the remaining tissue receives a decreased dosage to limit the side effects of the brachytherapy treatment. Another advantage of the proposed method is the use of the brachytherapy needles as imaging tools. Specifically, the imaging tools utilized are already in place because of the brachytherapy procedure. Therefore, the distortion and swelling of the gland due to the insertion of the needles are already accounted for.

This paper is structured as follows: section 2.1 outlines the mathematical background of EIT, specifically the execution of the Modified Newton Raphson algorithm. It is then followed by different sets of physical experiments in section 2.2. Two different objects were analyzed inside varying mediums: a solution of distilled water with sodium chloride as well as a soft gelatin medium. The discussion and conclusion is presented last in sections 4, 5.

## 2. Materials and Methods

### 2.1. Electrical Impedance Tomography

EIT begins by injecting an electrical current $I\left(\vec{x}\right)$ into a medium of interest ζ and observing the resulting electrical voltages on the boundary of the medium. The voltage distribution inside the medium is defined as $U\left(\vec{x}\right)$ and the boundary voltage readings are defined as $U_{b}\left(\vec{x}\right)$. Let the medium and resistance distribution be $\sigma \left(\vec{x}\right)$, where $\vec{x}$ is the voxel position within the medium. The goal of EIT is to vary the electric current on the boundary of the medium, ∂ζ, and from the resulting voltages, infer the internal resistance distribution $\sigma \left(\vec{x}\right)$ (Seo, 2012).

For the problem of EIT, a relationship between the electric field vector $E\left(\vec{x}\right)$ inside ζ and the voltage distribution $U\left(\vec{x}\right)$ can be established as:

(1) $$\begin{array}{l} -\nabla U\left(\vec{x}\right)=E\left(\vec{x}\right),\forall \vec{x}\in \zeta . \end{array}$$

Similarly, the electric current, $I\left(\vec{x}\right)$, can also be defined using the electric field vector:

(2) $$\begin{array}{l} I\left(\vec{x}\right)=\sigma \left(\vec{x}\right)E\left(\vec{x}\right),\forall \vec{x}\in \zeta . \end{array}$$

The electric current is injected on the boundary of the medium, ∂ζ. Using a normal vector on the boundary $n\left(\vec{x}\right)$ and boundary voxels $\vec{y}$, (2) can be written as:

(3) $$\begin{array}{l} I_{bound}\left(\vec{y}\right)=-\sigma \left(\vec{y}\right)\left[\nabla U\left(\vec{y}\right)\cdot \vec{n}\left(\vec{y}\right)\right],\forall \vec{y}\in \partial \zeta . \end{array}$$

It is assumed that the electric charges entering and leaving ζ are the same, thus the net charge equals zero:

(4) $$\begin{array}{l} \int_{\partial \zeta }I_{bound}\left(\vec{y}\right)\cdot \vec{n}\left(\vec{y}\right)da_{s}=0,\forall \vec{y}\in \partial \zeta , \end{array}$$

where *a*_*s*_ is the surface area of ∂ζ. Applying Maxwell's equation and the divergence theorem onto (4), the mathematical structure of EIT can be modeled by Poisson's Equation (Seo, 2012):

(5) $$\begin{array}{l} \nabla \left[\sigma \left(\vec{x}\right)\nabla U\left(\vec{x}\right)\right]=0,\forall \vec{x}\in \zeta . \end{array}$$

Equation (5) states that the gradient of $U\left(\vec{x}\right)$ is the direction in which electrons will flow as well as the gradient of voltage. The gradient of $U\left(\vec{x}\right)$ is multiplied with conductance, and the product gives the current flowing in ζ. And as defined by (4), the net charge inside ζ is zero, so the divergence of the current at $\vec{x}$ is also zero (Cheney and Isaacson, 1995).

The Dirichlet Boundary condition specifies the voltage, $U\left(\vec{x}\right)$, on the boundary of the medium (Seo, 2012):

(6) $$\begin{array}{l} U(\vec{y})]=t_{b},\forall \vec{y}\in \partial \zeta , \end{array}$$

where *t*_*b*_ represents boundary voltage. Likewise, the Neumann Boundary condition constrains the electric current from (3), given a known and predefined current vector *g*_*c*_ as:

(7) $$\begin{array}{l} U\left(\vec{y}\right)\cdot \vec{n}\left(\vec{y}\right)=g_{c},\forall \vec{y}\in \partial \zeta . \end{array}$$

In most cases, the amount of unknown parameters, $\sigma \left(\vec{x}\right)$, exceeds the known parameters, $U_{b}\left(\vec{x}\right)$, making the problem of EIT notably ill-posed (Seo, 2012; Sbarbaro et al., 2015). To solve the problem of EIT, an iterative procedure is usually employed using a forward solution and an inverse solution.

The forward solution determines $U_{b}\left(\vec{x}\right)$ when electrical current is injected upon the boundary of the medium, ∂ζ. In the case of the forward solution, the internal resistance distribution, $\sigma \left(\vec{x}\right)$, of ζ is known. The boundary voltages $U_{b}\left(\vec{x}\right)$ are then functions of the injected electrical current as well as the resistance distribution:

(8) $$\begin{array}{l} U_{b}\left(\vec{y}\right)=f\left(I\left(\vec{y}\right),\sigma \left(\vec{x}\right)\right),\forall \vec{y}\in \partial \zeta \wedge \vec{x}\in \zeta . \end{array}$$

On the contrary, the inverse solution determines $\sigma \left(\vec{x}\right)$ when the boundary voltages $U_{b}\left(\vec{x}\right)$ are known. Solving for the resistance distribution $\sigma \left(\vec{x}\right)$ is then a function of the injected electrical current as well as the boundary voltages:

(9) $$\begin{array}{l} \sigma \left(\vec{x}\right)=f^{-1}\left(I\left(\vec{y}\right),U_{b}\left(\vec{y}\right)\right),\forall \vec{y}\in \partial \zeta \wedge \vec{x}\in \zeta . \end{array}$$

Both (8) and (9) are executed in an iterative fashion in order to solve the problem of EIT. There are various different approaches for the forward and inverse solutions. These algorithms range from Single Value Decomposition, to Modified Newton Raphson and Optimal First Order Approximation. The approach outlined in this paper is explained in sections 2.1.1 and 2.1.2. A comprehensive review of existing inverse algorithms is summarized in Yang and Peng (2003); Lionheart (2004); Holder (2005); Neumayer et al. (2011); Canali et al. (2016); Fonseca et al. (2016); Rymarczyk et al. (2019); Gomes et al. (2020); Jauhiainen et al. (2020), and Padilha Leitzke and Zangl (2020).

**Figure 1:**
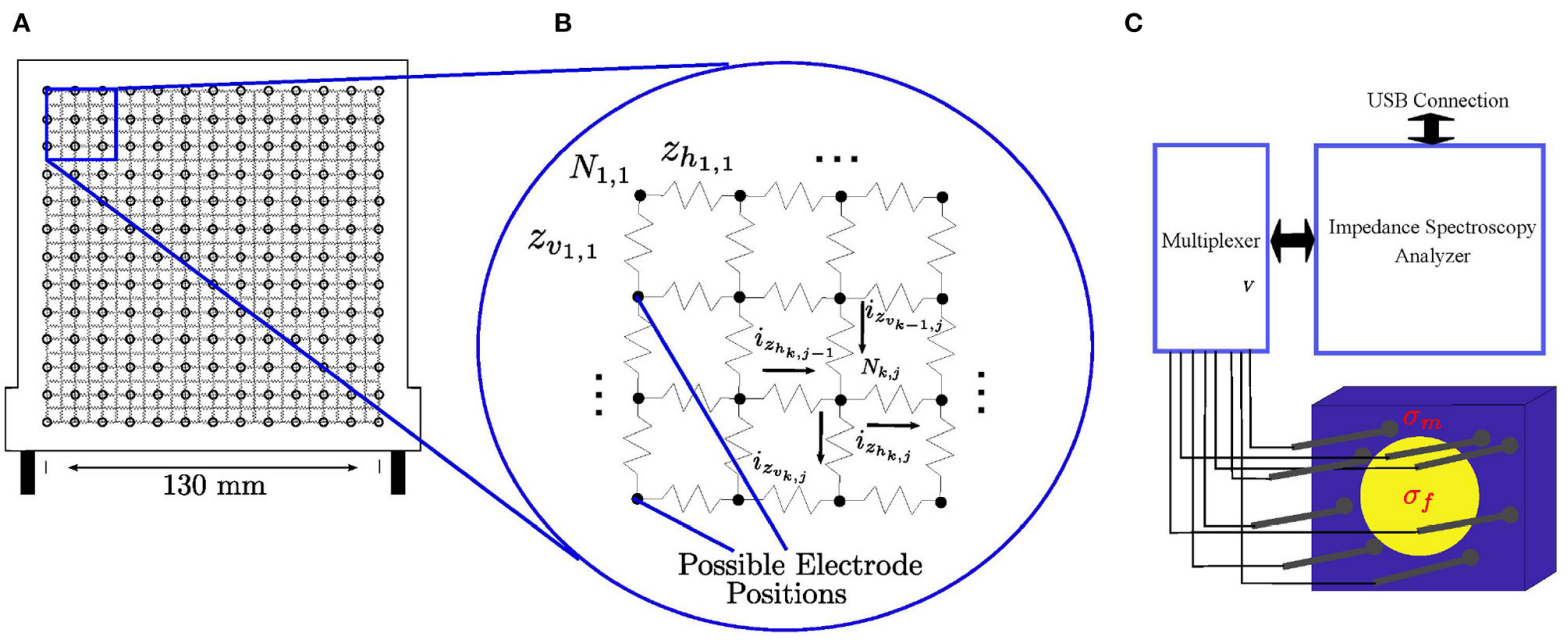
**(A)** The brachytherapy needles are inserted into the medium via a standard brachytherapy template. The resistor mesh is overlaid on top of the template where each node is an insertion point into the medium, which is represented by the resistor grid. In **(B)**, the needles act as electrodes once inserted into the electrical circuit to inject current and measure voltage. In **(C)**, multiple brachytherapy needles are used to image a medium (σ_*m*_) with cancerous tissue inside (σ_*f*_).

To calculate the forward and inverse solution, the medium ζ is defined as a square resistor mesh of finite size whose resistance values are to be determined. To access the medium, the brachytherapy needles are inserted through a standard brachytherapy template. The resistor grid is overlaid with the brachytherapy template where each node of the template represents a possible insertion site into the medium, which is represented by the resistor grid, as demonstrated by Figures 1A,B. For any given resistor grid, there are 2(*s*^2^ − *s*) amount of resistors where *s* is the number of nodes in the horizontal as well as the vertical direction. Correlating that to the physical brachytherapy template, each resistor then has a representative size of 130 mm/(*s* − 1) × 130 mm/(2*s* − 1) relative to the template. Once inserted into the medium, the needles act as electrodes for current injection and voltage measurement within the electrical circuit mesh as demonstrated by Figure 1B. An illustration of the process is shown in Figure 1C. where the needles are inserted into the medium to image the cancer growth within.

#### 2.1.1. Forward Solution

In the circuit of Figure 1B, each resistor is labeled as either *z*_*v*_*k,j*__ or *z*_*h*_*k,j*__, depending on if it is a vertical or horizontal resistor. Indexes 1 ≤ *k* ≤ *s* and 1 ≤ *j* ≤ *s* determine the coordinate position of each node in the electrical circuit. Similarly, the current flowing in each branch is labeled as *i*_*v*_*k,j*__ or *i*_*h*_*k,j*__, depending on if it is a vertical or horizontal branch. The nodes themselves are labeled as *N*_*k,j*_.

After the resistor circuit model is established, the sum of current entering and leaving a node is zero (Svoboda and Dorf, 2013):

(10) $$\begin{array}{l} \sum i=0. \end{array}$$

By applying (10) to any arbitrary node in the electric circuit of Figure 1 gives:

(11) $$\begin{array}{r} i_{z_{h_{k,j-1}}}+i_{z_{v_{k-1,j}}}-i_{z_{v_{k,j}}}-i_{z_{h_{k,j}}}-I_{N_{k,j}}=0,\\ \text{With}\{\begin{array}{c} {\text{i}}_{z_{h_{k,0}}}{\text{ = i}}_{z_{h_{k,s}}}\text{= 0}\\ {\text{i}}_{z_{v_{0,j}}}{\text{ = i}}_{z_{v_{s,j}}}\text{ = 0} \end{array} \end{array}$$

where *I*_*k,j*_ is the current injected into the arbitrary node *N*_*k,j*_. Furthering (11) as a function of the voltages at each node *v*_*N*_*k,j*__ becomes:

(12) $$\begin{array}{l} \frac{v_{N_{k,j-1}}-v_{N_{k,j}}}{z_{h_{k,j-1}}}+\frac{v_{N_{k-1,j}}-v_{N_{k,j}}}{z_{v_{k-1,j}}}\\ -\frac{v_{N_{k,j}}-v_{N_{k,j+1}}}{z_{h_{k,j}}}-\frac{v_{N_{k,j}}-v_{N_{k+1,j}}}{z_{v_{k,j}}}=I_{N_{k,j}}\\ \text{With}\{\begin{array}{c} z_{h_{k,0}}{\text{ = z}}_{{\;}_{h_{s,j}}}\text{= }\infty \;\\ z_{v_{k,s}}{\text{ = z}}_{v_{0,j}}\text{ = }\infty \end{array} \end{array}$$

By applying (12) to every node in the resistor grid of Figure 1, a system of linear equations can be established with as many nodal equations as (12), as there are unknown nodal voltages, *v*_*N*_*k,j*__. In matrix form, this is represented as:

(13) $$\begin{array}{l} C^{*}V_{f}=I \end{array}$$

where $C^{*}\in {ℛ}^{s^{2}\times s^{2}}$ is the conductivity matrix constructed entirely of conductance values (inverse of resistor values). The matrix $V_{f}\in {ℛ}^{s^{2}\times 1}$ and $\text{I}\in R^{s^{2}\times 1}$ are vectors that hold all the nodal voltages as well as injected nodal currents in every node of the resistor grid, respectively. The latter is generally populated entirely of zero values except for two non-zero terms: +*I*_*N*_*k,j*__ and −*I*_*N*_*k,j*__, which represent the current injection sites, the positive current injected node and the negative exiting current node. It can be observed that all the values of **I** sum to zero as proof of (4).

To solve the system of linear equations and obtain the voltage **V**_**f**_, an inverse is required for C^*^. However, in its current form, det(C^*^) = 0 and the inverse cannot be computed. To remedy this, a node in the resistor grid has to be grounded i.e., a full row and column of the conductance matrix is set to zero. The Hadamard Product is used to perform this operation. The Hadamard Product is a mathematical matrix operation in which two matrices are multiplied together in an element wise fashion (Colbourn, 2014):

(14) $$\begin{array}{l} C=C^{*}\circ G. \end{array}$$

$\text{C}\in R^{s^{2}\times s^{2}}$ is non-singular and $\text{G}\in R^{s^{2}\times s^{2}}$ has a unit value for every term, except zeros for the column and row corresponding to the grounded node. The diagonal term **G**_*g,g*_ corresponding to the diagonal term of the grounded node in the conductivity matrix is set to $\frac{1}{C_{g,g}}$, where *g* is the index term in **C** counting from the first row until the row corresponding with the node that is to be grounded. The term $\frac{1}{C_{g,g}}$ is used to multiply with *C*_*g,g*_ in order to result with a product of exactly 1.

With the conductance matrix non-singular, the forward solution can be calculated using a simple matrix inversion.

(15) $$\begin{array}{l} V_{f}=C^{-1}I \end{array}$$

For every distinct injection pattern (variation of **I**), there is a distinct output of **V**_**f**_. The forward solution of EIT utilizes (15) to calculate various different voltage values via different current injection patterns in order to amass sufficient unique measurements to perform the inverse solution.

#### 2.1.2. Inverse Solution

The inverse solution is executed using the Modified Newton Raphson (MNR) algorithm. To start the algorithm, an initial resistance distribution is required. This is usually a vector of uniform resistance value. The approach is a deterministic method that iteratively updates the initial resistance distribution until the calculated voltages resemble the measured voltages (Yorkey et al., 1987). This iterative update is calculated as:

(16) $$\begin{array}{l} {\text{z}}^{k+1}={\text{z}}^{k}+\Delta {\text{z}}^{k}, \end{array}$$

where **z**^*k*^ ∈ ℝ^1×^^(2*s*^2^−2*s*)^ holds all the resistor values in iteration *k* and Δ**z**^*k*^ are the calculated update values to be added to **z**^*k*^ while **z**^*k*+1^ ∈ ℝ^1×^^(2*s*^2^−2*s*)^ is the updated resistance distribution at iteration *k*+1. The algorithm will be deemed complete once Δ**z**^*k*^ is below a predefined value or if the *k* iteration counter reaches a predefined value.

The solution depends on the initial resistance distribution, **z^0^**. From the work of Murai and Kagawa, it is determined that the solution is generally acceptable if: $|z^{0}-z_{t}|<10$ (Murai and Kagawa, 1985).

The catalyst of driving the iterative procedure (16) is calculating the Δ**z**^*k*^ term based on the least square error θ(**z**^*k*^) between the measured and estimated voltages as (Yorkey et al., 1987):

(17) $$\begin{array}{l} \theta \left({\text{z}}^{k}\right)=\frac{1}{2}{\left[\text{V}\left({\text{z}}^{k}\right)-{\text{V}}_{0}\left(\zeta \right)\right]}^{T}\left[\text{V}\left({\text{z}}^{k}\right)-{\text{V}}_{0}\left(\zeta \right)\right] \end{array}$$

The matrix **V** ∈ ℝ^*n*×*p*^ contains calculated voltage values derived from (15). Likewise, ${\text{V}}_{0}\in {\mathbb{R}}^{n\times p}$ are then respective measured voltages for *n* amount of voltage readings and *p* amount of current injection patterns.

To minimize the error, (17) is differentiated with respect to **z**^*k*^ and set equal to zero, i.e.,

(18) $$\begin{array}{ll} \frac{\partial \theta }{\partial {\text{z}}^{k}} & ={\frac{\partial \text{V}}{\partial {\text{z}}^{k}}}^{T}\left[\text{V}-{\text{V}}_{0}\right]=0,\text{or} \end{array}$$

(19) $$\begin{array}{ll} \theta^{\prime } & ={\left[{\text{V}}^{\prime }\right]}^{T}\left[\text{V}-{\text{V}}_{0}\right]=0. \end{array}$$

In the above, the term **V**′ = ∂**V**/∂**z**^*k*^ ∈ ℝ^*n*×2^^*s*^^^2^−2*s*^ is the Jacobian matrix, describing the rate of change of the voltage values with respect to each of the resistors (Yorkey et al., 1987). In matrix form, the Jacobian is displayed as:

(20) $$\begin{array}{l} \left[{\text{V}}^{\prime }\right]=\left[\begin{array}{cccc} \frac{\partial V_{1}}{\partial z_{1}} & \frac{\partial V_{1}}{\partial z_{2}} & \ldots & \frac{\partial V_{1}}{\partial z_{2s^{2}-2s}}\\ \frac{\partial V_{2}}{\partial z_{1}} & \frac{\partial V_{2}}{\partial z_{2}} & \ldots & \frac{\partial V_{2}}{\partial z_{2s^{2}-2s}}\\ \vdots & \vdots & \ddots & \vdots \\ \frac{\partial V_{n}}{\partial z_{1}} & \ldots & \ldots & \frac{\partial V_{n}}{\partial z_{2s^{2}-2s}} \end{array}\right]. \end{array}$$

In its current state, (19) is a non-linear function of **z**. A Taylor Series Expansion is performed on (19) about an arbitrary point, **z** = **z**^*k*^ so that (19) can then be rewritten as:

(21) $$\begin{array}{l} \theta^{\prime }\approx \theta^{\prime }\left({\text{z}}^{k}\right)+\theta^{\prime \prime }\left({\text{z}}^{k}\right)\Delta {\text{z}}^{k}. \end{array}$$

The term θ″ = ∂^2^**V**/∂**z**^2^ is also known as the Hessian Matrix (Yorkey et al., 1987). It can be approximated as:

(22) $$\begin{array}{l} \theta^{\prime \prime }\approx {\left[{\text{V}}^{\prime }\right]}^{T}\left[{\text{V}}^{\prime }\right]. \end{array}$$

To find a solution for Δ**z**^*k*^, (19) and (22) are taken and substituted into (21) to yield:

(23) $$\begin{array}{l} \Delta {\text{z}}^{k}=-{\left\{{\left[{\text{V}}^{\prime }\left({\text{z}}^{k}\right)\right]}^{\text{T}}{\text{V}}^{\prime }\left({\text{z}}^{k}\right)+\lambda \text{W}\right\}}^{-1}\left[{\text{V}}^{\prime }\left({\text{z}}^{k}\right)\right]\left[\text{V}\left({\text{z}}^{k}\right)-{\text{V}}_{0}\right]. \end{array}$$

**Figure 2:**
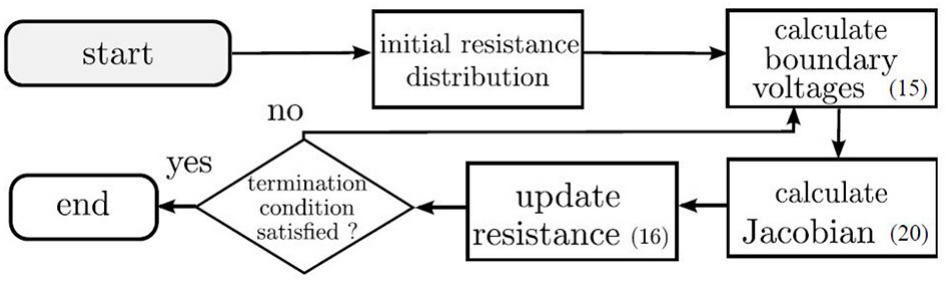
The MNR algorithm iteratively updates the initial resistance distribution until the calculated voltages converge to the measured voltages (Tan and Rossa, 2020).

The term Δ**z**^*k*^ can be entered into (16) to obtain the updated resistance distribution. In (23), the Marquadt method is utilized to mitigate the ill-conditioning of the inverse (Marquardt, 1963; Yorkey et al., 1987). The matrix, **W**, is an identity matrix and λ ∈ ℝ^+^ → 0 is a scalar. Multiplied together, the two terms prevent the system from reaching singularity. For this paper, the value of λ was selected to be between 1 × 10^−8^ and 1 ×10^−9^. The termination condition is set at 3 iterations of the MNR algorithm, in which the change in Δ**z**^*k*^ has stabilized. The overall procedure can be illustrated in the flowchart of Figure 2.

### 2.2. Experimental Setup

To test the feasibility of EIT in brachytherapy using needles as imaging tools, various different sets of experiments are conducted. A general diagram outlining the setup of the experiment is displayed in Figure 1C. The impedance spectroscopy analyzer is connected to a multiplexer which is connected to the needle electrodes. The impedance spectroscopy analyzer is able to inject electrical current and measure resulting voltages through the Eliko Quadra graphic user interface (Min et al., 2018). The electrodes are inserted into the medium through the brachytherapy template. Two different types of EIT imaging was executed for this study: absolute EIT imaging and frequency difference EIT. Absolute EIT imaging injects electrical current at a fixed frequency of 1 kHz. Frequency difference injects varying frequencies. The resulting voltages of are then subtracted from one another and then used in the reconstruction algorithm (Seo et al., 2008). The injected current is using a square wave pattern. The voltage measurement and current injection patterns utilized is the adjacent method (Rajaguru et al., 2013).

For all sets of experiments, eight 18-gauge standard brachytherapy needles with a length of 200 mm were used in each test as the EIT electrodes. The needle electrodes were connected to an impedance spectroscopy analyzer, specifically, the Eliko Quadra (Min et al., 2018). The spectroscope injects different current patterns and measures the resulting voltages through the eight attached needles. The resulting voltage measurements were imported into the custom MNR scripts.

For the experiments conducted in this study, the brachytherapy needles were inserted manually to the exact depth of the inclusions being observed. In real medical applications, a separate imaging modality (i.e., ultrasound) would be required to guide the insertion of the brachytherapy needles. It is a common practice to use an ultrasound imaging modality to guide brachytherapy needles, and its use has extended to various cancer treatment procedures such as prostate, cervix, and anal canal cancers (Banerjee et al., 2017). In needle insertion applications, the needle is assumed to travel in a straight path. However, this may not always be the case in real applications. As the needle is inserted and it interacts with the medium, it may steer away from the intended trajectory, especially in the case of bevel-tipped needles (Rossa and Tavakoli, 2017). There are various closed-loop needle steering systems that can mitigate needle deflection as the insertion takes places. A comprehensive review of closed-loop needle steering systems can be found in Rossa and Tavakoli (2017). To track the needle insertion, 2-dimensional ultrasound images of the needle tip is often generated. Using information from the ultrasound images, the needle shape, deflection and trajectory can be planned to achieve a desired path and depth (Carriere et al., 2016).

The experiments were conducted with an adjacent current injection and voltage pattern with a magnitude of 15 mA (Rajaguru et al., 2013). With a total of eight electrodes, there were 40 unique measurements for each experiment. It is important to note that the needles used as current injection sites were not used for voltage measurements, which attributed to the total amount of five measurements in each of the eight injection patterns. The tomography image was constructed based on an electrical mesh with 1,300 resistors. The standard brachytherapy template used has a 13 needle insertion points both vertically and horizontally. The number of resistors in the grid is an even multiple of the number of insertion points, i.e., 26 nodes vertically and horizontally, such that all the needle insertion points match the location of a given node in the grid. With a resistor grid of size 26 ×26, there are 1,300 resistors as a result.

#### 2.2.1. Scenarios 1A and 1B: Large High Conductive Object in Distilled Water and Sodium Chloride

**Figure 3:**
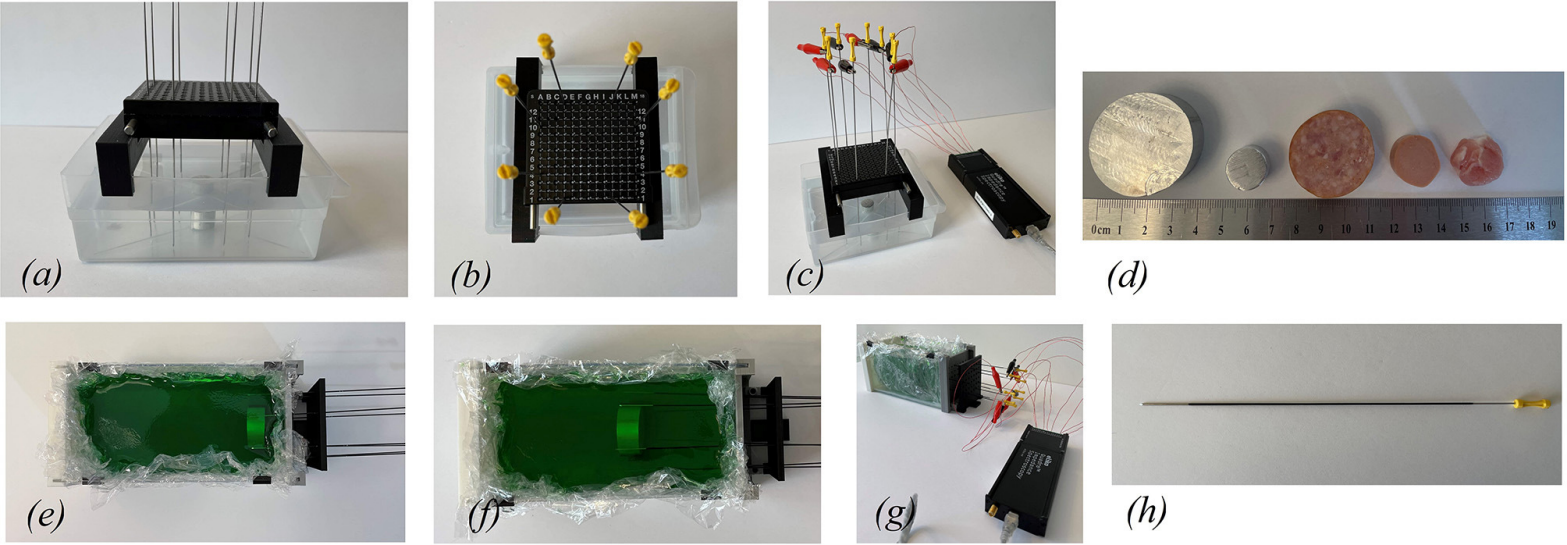
**(a)** Side view of the water tank setup is displayed. **(b)** A standard brachytherapy template was used. **(c)** The needles are connected to an impedance spectroscopy analyzer. **(d)** Various different inclusions were used for the experiments. **(e)** The cylinder placed at 15 mm from the needle insert plane. **(f)** The inclusions were placed at 76 mm from the needle insert plane. **(g)** The needles are connected to an impedance spectroscopy analyzer. **(h)** The needles are coated with an insulation compound.

The first set of experiments were performed inside a 135 × 115 mm container with a height of 55 mm filled with 0.5 L of distilled water mixed with 8.5 g of sodium chloride, as displayed in Figures 3a–c. A standard brachytherapy template for insertion of the needles was used for all tests in this paper. An aluminum cylinder with a diameter of 38 mm and a height of 17 mm was used, see Figure 3d. The tests are performed with the cylinder situated in the middle, corresponding to scenario 1.A. as well as the top of the container, corresponding to scenario 1.B. The needles are inserted through the brachytherapy template and are positioned around the aluminum cylinder.

#### 2.2.2. Scenarios 1C and 1D: Small High Conductive Object in Distilled Water and Sodium Chloride

The second set of tests used a small aluminum cylinder with a diameter of 16 mm and a height of 13 mm, as shown in Figure 3d. The same rectangular container from Scenarios 1.A. and 1.B. was used. Two tests were performed with the small aluminum cylinder. In scenario 1.C., the cylinder is located in the middle and in scenario 1.D., the cylinder is located toward the bottom of the container.

#### 2.2.3. Scenarios 2A and 2B: Large High Conductive Object in Gelatin

The third set of experiments are performed in a larger rectangular container of 180 × 95 mm and height of 100 mm. The larger container was filled with gelatin to mimic soft human tissue. Similar to scenarios 1A and 1B, the same large aluminum cylinder was used for this set of experiments. In scenario 2A, the cylinder was first placed at 25 mm off the base of the container and 15 mm away from the needle entrance plane. In scenario 2B, the cylinder was placed 25 mm off the base and 76 mm deep from the needle entrance plane. The top view of the gelatin setup is displayed in Figures 3e,f. The gelatin setup connected to the impedance spectroscopy analyzer and the coated needle are displayed in Figures 3g,h.

#### 2.2.4. Scenario 3: Bovine Gelatin Sample

In this scenario, soft bovine meat was used instead of the aluminum cylinder. The bovine meat was placed 25 mm off the base of the container and 76 mm deep from the needle entrance plane. The bovine meat itself has a cylindrical shape with a diameter of 32 mm and a height of 17 mm. The bovine meat can be seen in Figure 3d.

#### 2.2.5. Scenario 4: Chicken Gelatin Sample

Similar to scenario 3, this experiment uses soft chicken meat as the inclusion casted inside gelatin. The container size is consistent with the rest of the experiments. The chicken meat itself has a cylindrical shape with a diameter of 19 mm and a height of 17 mm. The chicken meat can be seen in Figure 3d.

#### 2.2.6. Scenario 5: Pork Loin Gelatin Sample

In the last scenario, a sample piece of pork loin is obtained. It is casted inside the gelatin container as per the previous experiments. The pork loin tissue sample has width of 20 mm and, length of 18 mm and height of 14 mm. The pork loin sample can be seen in Figure 3d.

For the last scenario setup, ultrasound imaging was conducted on the gelatin setup. The ultrasound machine, Verasonics Vantage 64LE, was utilized. The ultrasound probe was positioned on top of the exposed gelatin. Vertical ultrasound images were obtained on the pork loin that was casted inside the gelatin. The comparison of the EIT images to the ultrasound image is discussed in section 4.

## 3. Results

**Figure 4:**
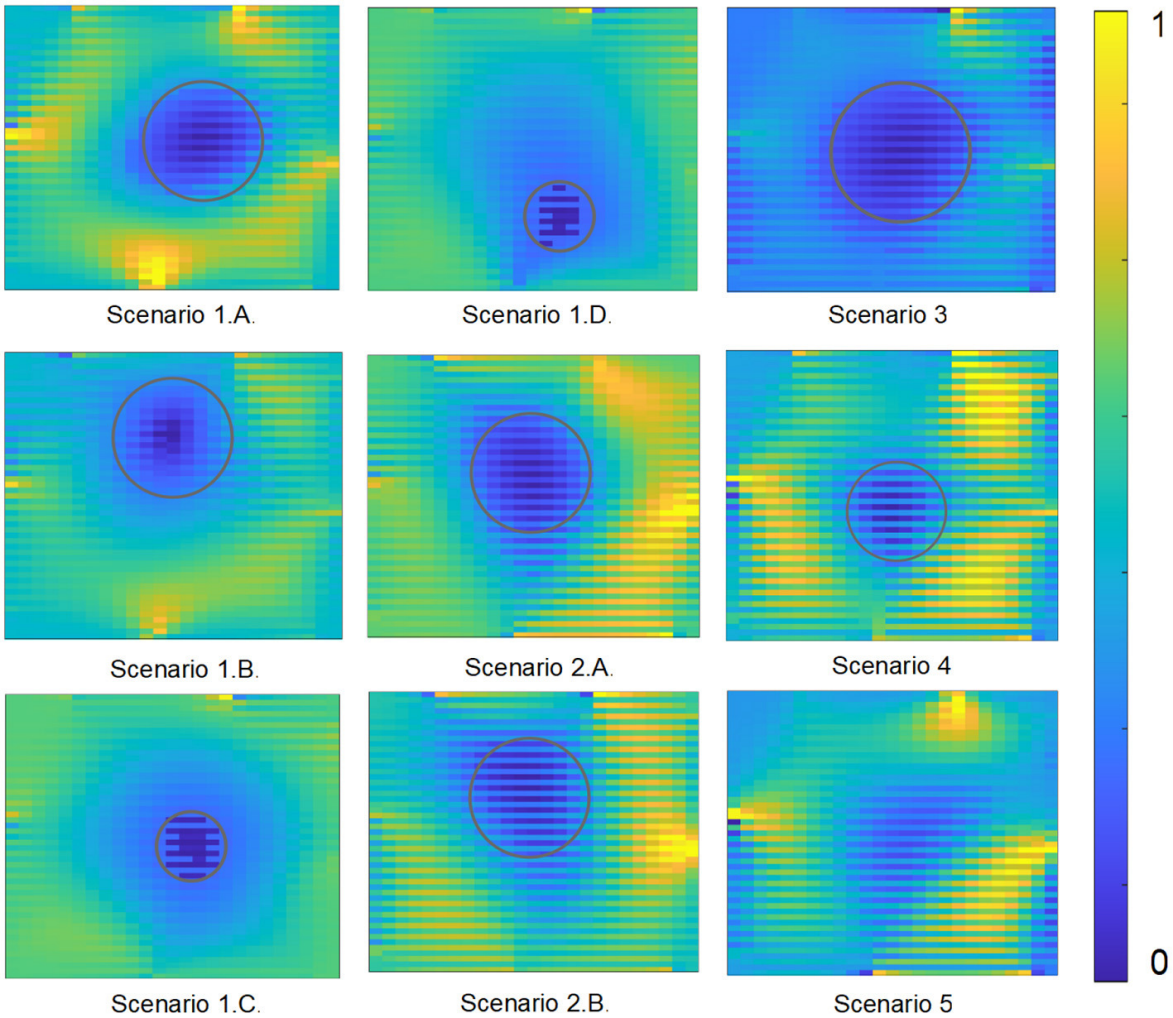
The results are displayed for each of the different experimental scenarios with the resistance values normalized to range from 0 to 1 in order to display a consistent color contrast.

A total of six different tests were conducted in different mediums while using different observed objects. The output tomography images for each of the different test scenarios are displayed in Figure 4.

## 4. Discussion

In Figure 4, the gray outlined circle indicate the inclusions within the EIT images for all the resulting scenarios except for scenario 5, since it is an irregular shaped object. In scenarios 1A and 1B, EIT is able to clearly delineate the cylinder from the surrounding medium. The initial resistance distribution to launch MNR was 5 Ω. There is slight noise in the system as can be observed from the discoloration in the background. This is due to the brachytherapy needle as the location of the noise correspond to the location of the insertion points.

In scenarios 1C and 1D, the object was imaged while situated in the center of the container as well as near the bottom of the container. Similar to the tests from the previous scenarios, the algorithm was initialized with a 5 Ω distribution. As can be observed from the image results, the object is clearly discriminated against its background medium. This is important as EIT is able to delineate smaller objects within the same medium.

In scenarios 2A and 2B, the object was placed at different depths along the container. Like before, EIT was able to clearly identify the object by detecting the resistance distribution inside the gelatin medium. It is important to note that gelatin is less conductive than distilled water mixed with sodium chloride making it more difficult to obtain EIT images. The initial resistance distribution to launch MNR for the gelatin medium was 60 Ω. The results obtained were successful in delineating the large aluminum cylinder inside gelatin.

For scenario 3, the bovine meat sample was placed at 76 mm from the needle entrance plane. Similar to scenarios 2A and 2B, the inclusion was able to be delineated clearly against the background environment. The relative size of the bovine meat sample is consistent with the large aluminum cylinder.

In scenario 4, a smaller meat sample was casted inside the gelatin. Specifically, the chicken meat of diameter 19 mm was placed at the same depth of 76 mm inside the gelatin. The EIT implementation was successful in separating the conductivity of the chicken sample from the surrounding gelatin tissue.

In the last scenario, a small piece of pork loin was obtained and placed deep inside the gelatin. Similar to previous experiments, the inclusion is clearly defined against its background conductivity.

These results are critical as it proves the effectiveness of the brachytherapy needles in reaching depths far beneath the surface. The brachytherapy needles in this case, are able to penetrate to the exact depth of the object and record electrical information at the precise location. This is crucial in the case of prostate cancer as the tissue structure is located several inches within the body from all sides. Using brachytherapy needles, the prostate can be reached and EIT can be performed within the body.

**Figure 5:**
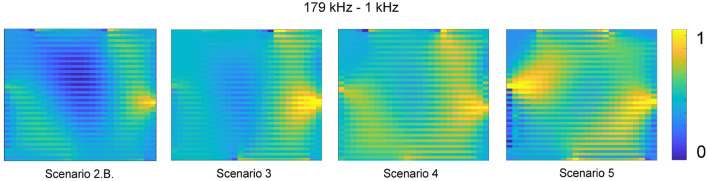
The results for the soft biological inclusions imaged using different frequency injections are displayed with the resistance values normalized to range from 0 to 1 in order to display a consistent color contrast.

In addition to utilizing absolute difference EIT imaging, different frequencies were also explored in constructing the tomographic images. The Eliko Quadra is capable of producing 15 different frequencies from 1 up to 349 kHz (Min et al., 2018). A few sample EIT images created using frequency difference, specifically the difference between 179 and 1kHz, is displayed in Figure 5. It is determined that in the proposed gelatin samples, absolute imaging performed better than frequency difference imaging.

**Figure 6:**
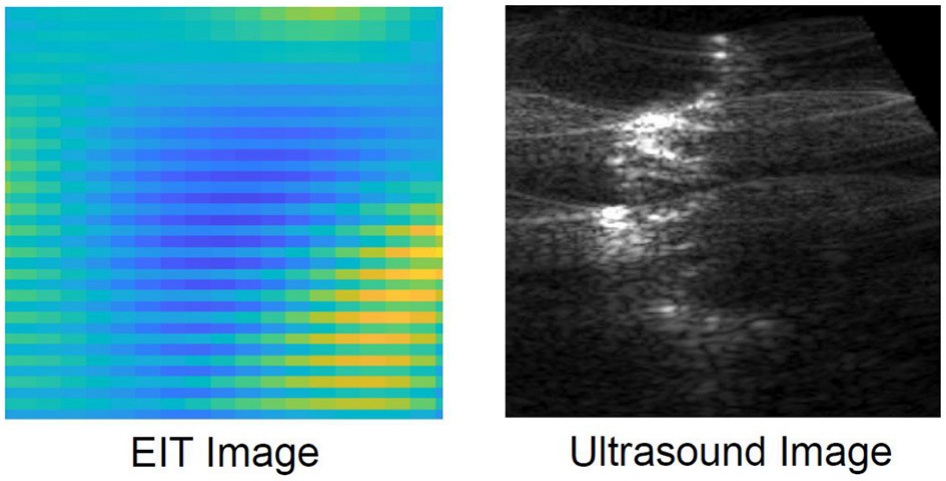
The EIT image for scenario 5 is compared against its ultrasound image.

The inclusion under observation in scenario 5 was an irregular biological tissue with an undefined shape. It is not a circular tissue, like in scenarios 1–4. Therefore, it is challenging to define the inclusion for comparison against the reconstructed EIT image. As a result, the reconstructed EIT images for scenario 5 was compared with another common imaging modality. An ultrasound image of the pork loin was produced by placing the probe directly above the gelatin. The EIT image and ultrasound image clearly identify the inclusion within the gelatin as shown in Figure 6.

## 5. Conclusion

Temporary brachytherapy, also known as HDR, is an effective radiation treatment procedure for prostate cancer. Unlike whole gland radiation treatment, HDR is able to precisely target a specific tumor growth inside the prostate (American Cancer Society, 2020). This targeted tumor is usually the epicenter and driver of the malignant cancer growth. To maximize the performance of HDR, an effective and high contrast imaging procedure is required. Electrical impedance tomography is proposed in this paper for the imaging procedure as it is relatively inexpensive and the nature of its algorithm provides high contrast tomography outputs. Brachytherapy needles are employed as the electrodes for the process of EIT. Having needle electrodes allow the voltage readings to penetrate far beneath the skin surface. This reduces potential noise and provides stronger electrical readings not otherwise achievable with traditional EIT surface electrodes (Meroni et al., 2017).

In this paper, experiments are conducted using different mediums. A total of eight brachytherapy needles inserted in the medium via a standard brachytherpay guide template were connected to an impedance spectroscopy analyzer. A large and a small aluminum cylinder were placed in distilled water mixed with sodium chloride. The resulting tomography images were able to delineate the object from the surrounding environment. Gelatin tests were also conducted to mimic soft human tissue and recreate a brachytherapy setup. The large aluminum cylinder was placed at different depths inside a long rectangular container. In addition, soft bovine meat, chicken meat and pork loin were used as inclusions as well to replicate a realistic brachytherapy setup. The needles penetrated the gelatin setup from a horizontal entrance on the side. The readings were recorded inside the gelatin and EIT was able to successfully characterize the aluminum cylinder from the gelatin background. Frequency difference EIT imaging was conducted alongside absolute EIT imaging and it was determined that absolute imaging performed superior in terms or tomographic output. The final EIT image of scenario 5 was also compared with an ultrasound image to verify the correct size and spatial location of the pork loin.

The results indicate the feasibility of utilizing multiple brachytherapy needles for the purpose of conducting EIT from within the human prostate. The needles can access a depth within the medium that surface electrodes cannot. This is an advantage over traditional EIT and it can highly impact the imaging procedure for internal radiation treatment. Further refinements will follow to enhance the tomography images. This includes exploring different brachytherapy needle setups such as coating the needles with different insulation compounds as well as inserting more than eight needle electrodes. The average amount of needles utilized for brachytherapy is 18.8 needles (Steggerda et al., 2010) and thus there is potential to utilize more than eight needles to conduct EIT. With more electrodes in the medium, it is theorized that the problem of EIT will be less ill-posed as there are more measured voltages and the reconstructed images will be enhanced as a result. Furthermore, tests on different mediums will be explored. This includes creating different conductive mediums as well as *ex-vivo* biological tissue to perform the tests in. Research into controlling the signal to noise ratio will have to be performed when experimenting with *ex-vivo* biological tissue. In addition, a deep analysis into observing the conductivity variance between biological malignant and benign tissues will also be explored. The potential to complement the EIT procedure using additional imaging techniques will also be explored.

EIT holds great promise in brachytherapy as it provides critical tomographic information within the prostate. When EIT is coupled with HDR, the treatment for malignant tissue within the prostate can be exceptionally effective. It is also theorized that the needle insertion location can be optimized, ie. needles inserted closer together, needles inserted farther apart, needle location in relation to the focal mass. Once optimized, it is theorized to provide better readings in terms of signal to noise ratio and, as a result, develop a more precise tomographic image. Future work will focus on using real-time EIT to guide a brachytherapy needle toward a predefined target via robotic assistance.

## Data Availability Statement

The datasets presented in this article are not readily available because, the dataset is available for the corresponding author only. Requests to access the datasets should be directed to hao.tan1@ontariotechu.net.

## Author Contributions

HT and CR conceived and planned the study as well as the various experiments. HT carried out the experimental studies with contributions from CR. All authors performed the analysis, interpretation, discussion of the experimental results, and approved the final submitted paper. HT wrote the manuscript with contributions from CR.

## Conflict of Interest

The authors declare that the research was conducted in the absence of any commercial or financial relationships that could be construed as a potential conflict of interest.
